# Reflections on connections

**DOI:** 10.1007/s10329-023-01059-8

**Published:** 2023-03-03

**Authors:** Martha M. Robbins

**Affiliations:** Department of Primate Behavior and Evolution, Max Planck Institute for Evolutionary Anthropology, Leizpig, Germany

## Introduction

**Fig. 1:**
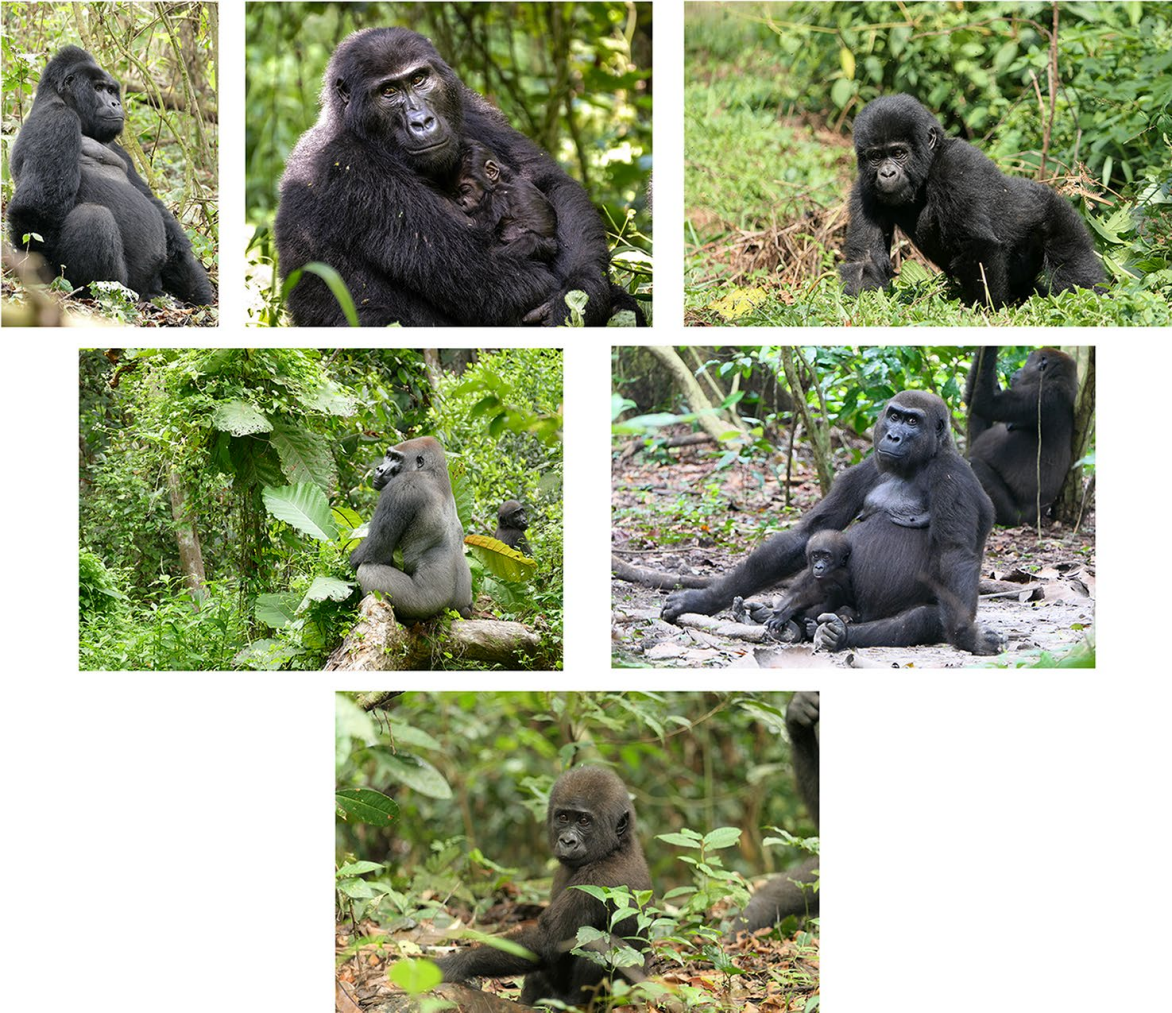
*From top to bottom* Silverback mountain gorilla, adult female mountain gorilla with infant, infant mountain gorilla, silverback western gorilla, adult female western gorilla with infant, infant western gorilla. Photo credits: Martha M. Robbins/MPI-EVA

Connections among people are part of everyday life for all of us, but they also are a key component for the success of primatology. As we all continue to seek normality after the disruption that the COVID-19 pandemic caused on individual, local, and global levels, it is commonplace to appreciate the value of connections we have among one another. Furthermore, 2023 is a time for me to personally reflect on connections because it is the 25th anniversary of the research project I have directed on the Bwindi mountain gorillas (*Gorilla beringei beringei*) in Uganda [and not as notable a time period, but 18 years since I started a project studying western gorillas (*Gorilla gorilla gorilla*) in Loango National Park, Gabon]. Such an anniversary leads to reflections on connections that were crucial for the maintenance of long-term field sites (Fig. 1). Primatology rests on three components of connections: among primates, among disciplines, and among people. Here I offer some reflections on connections, perhaps none novel, with the aim to synthesize topics that can help drive primatological research forward and ensure we have primates in the future.

## Connections among primates

The evolution of sociality, which rests on the invisible ties among individuals, has intrigued primatologists since the birth of the discipline (Hinde 1976; Itani 1977), and continues to be a driving force in our research. The patterning of connections of individuals, or social interactions, results in the social structure for a particular species (Hinde 1976; Schülke et al. 2022). The variations in patterns of sociality in group-living animals and the challenge of understanding what causes these variations are what led me to become a primate behavioral ecologist. Research in primatology has focused on several aspects of connections, including the search for a unified framework to describe the variation in social structure among primates, the role of dominance in sociality and reproductive success, the value of affiliative relationships or social bonds, and social learning resulting in animal culture.

Examining the linkages between ecological conditions and variation in primate social behavior has been a foundational aspect of primatology (Crook and Gartlan 1966; Clutton-Brock and Harvey 1977). This included the development of “socioecological models,” with the goal of creating a unified framework to predict patterns of agonism, affiliation, kinship, and dispersal (e.g. Sterck et al. 1997; van Schaik 1989; Wrangham 1980). Over time, empirical data have revealed many contradictions to the predictions of various iterations of the socioecological model, but the overall concept of testing hypotheses of how ecological variation influences social behavior still has utility (Clutton-Brock and Janson 2012; Koenig et al. 2013). Perhaps the variation observed within and among species, the complexity of both ecological conditions and reproductive competition, as well as the intricacies of social relationships render it challenging to create a unified framework. For example, inter- and intra-species comparisons of gorilla populations have shown considerable spatial and longitudinal variation in diet, association patterns, and social structure (Ganas et al. 2004; Robbins and Robbins 2018; Robbins et al. 2022; Young and Robbins 2023). We can aspire to see emergent patterns of primate social structure through more detailed studies of individual species, both well studied and little known, as well as through comparative studies (e.g. Schülke et al. 2022). Examining social complexity may also be a way forward; however, there is ongoing debate about how to define, measure, and compare social complexity among species, as well as whether it is simply a conceptual framework for describing diversity of social systems or an actual property of animal societies (Bergman and Beehner 2015; Fischer et al. 2017; Kappeler 2019).

Agonistic behavior is part of the connections in group-living animals driven by competition for limited resources. The formation of dominance relationships and hierarchies is a means of regulating competition among individuals (Rowell 1974). Dominance relationships and priority-of-access models have long been key features in describing social interactions and correlates of reproductive success in primates (e.g. Majolo et al. 2012; Shivani et al. 2022). More recently, an emphasis has been placed on power, or the ability to exert control over other individuals, which addresses many of the limitations of focusing only on dominance (Lewis 2002, 2022). Additionally, studies have begun to focus on intersexual dominance and power relationships (Davidian et al. 2022; Kappeler et al. 2022; Koenig et al. 2022; Young et al. 2017), which seems logical given that males and females co-reside in social groups and need to balance their conflicting interests. Rather than using different theoretical frameworks for examining relationships within males or females, these studies show the value of integrating both sexes and competition for various resources into a single model (Kappeler et al. 2022). Such an approach holds potential for further understanding the causes and consequences of group living and the diversity of social structure.

Group living is assumed to provide net benefits due to individuals associating with conspecifics, but the benefits may vary among individuals depending on their level of social integration (Hinde 1976; Silk 2007). The degree to which individuals are integrated into their social group has been measured by differing metrics, including the degree of affinitive interactions or associations maintained by social proximity, affiliative relationships including grooming, and social bonds, which are defined as strong, equitable, and enduring relationships (e.g. Ostner and Schülke 2018; Silk et al. 2013). Variation in within-group sociality has been correlated with offspring production and survival as well as adult survival in many species (Ostner and Schülke 2018). Some studies have found that having a few strong, enduring social bonds may be beneficial in some species (e.g. Silk et al. 2009; 2018), whereas others have shown the benefit of having many weak social bonds (McFarland et al. 2017). Further research should focus on the mechanisms linking sociality to fitness (Ostner and Schülke 2018) as well as the number of both weak and strong ties (Schülke et al. 2022).

In addition to genetic and environmental influences on social behavior, social learning enables individuals living in groups to observe and learn from one another, which sometimes leads to culture (Whiten and de Waal 2018; Whiten 2021). Many studies of culture in primates have relied on the method of exclusion to provide evidence for cultural variation in them (Perry 2011; Robbins et al. 2016; van Schaik et al. 2003; Whiten et al. 1999). However, Schuppli and van Schaik (2019) argue that this approach has limitations, and they suggest instead that focusing on counting socially learned traits will reveal large cultural diversity. Other approaches used to show evidence of social learning and cultural transmission include social network analysis (Hobaiter et al. 2014) and experiments in captive settings (van de Waal et al. 2013). Brakes et al. (2021) suggest that cultural traits contribute to the survival, reproduction, and population structure of many species, and should be integrated into conservation management strategies.

## Connections among disciplines

Primatology, the study of primates, sits at an intersection with many disciplines, including anthropology, psychology, zoology, and ecology. Therefore, primatology is inherently interdisciplinary, yet recent synergies among fields has helped advance our understanding of the causes and consequences of primate sociality.

Since the 1990s, genetic analysis, namely through genotyping, has greatly expanded our understanding of reproductive success and reproductive skew, kinship and relatedness, dispersal patterns, and population structure of wild primate populations (e.g. Arandjelovic and Vigilant 2018; Langergraber et al. 2009; Vigilant et al. 2015; Guschanski et al. 2008). In recent years, advances in techniques have enabled whole genome sequencing from non-invasively collected samples, which significantly increases the potential for genomic studies of wild populations (Snyder-Makler et al. 2016). Studies of genome-wide variation are providing insights into functional traits such as high-altitude adaptations and dietary patterns (Guevara et al. 2021; Chiou et al. 2022).

The interface between disease and primate sociality is receiving increasing research attention. The risk of disease to primate populations, particularly due to increased human interactions in primate habitats, has been at the forefront of conservation threats in recent decades (e.g. Gilardi et al. 2015). From the perspective of sociality, increasing evidence has revealed that the social environment may affect disease risk, mortality, and reproductive success in primates and other mammals (Snyder-Mackler et al. 2020). Additionally, the relationship between social behavior and microbiome appears to be an intriguing dynamic (e.g. Archie and Tung 2015).

Perhaps the most important connection among disciplines is between primatology and conservation biology. The majority of primate species are faced with the threat of extinction, with 75% having declining populations due primarily to habitat loss and degradation, hunting, and disease (Estrada et al. 2017). These threats have increased in intensity in the past decades, as has the impact of climate change (Chapman and Peres 2021).

An underutilized method in primate conservation is modeling that uses empirical data to estimate the impact of different scenarios of conservation interventions (e.g. Imong et al. 2016; Dobson et al. 2019). Additionally, conservation projects often do not include an evaluation of their effectiveness, which limits our understanding of what works and what does not (Junker et al. 2020). Most conservation research typically relies on long-term data or spatially widespread data, which may not be available for all species or ecological variables (Chapman and Peres 2021). For example, the consequences of climate change on large mammal populations are complex (Bernard and Marshall 2020), with studies indicating effects such as a decline in fruit availability (Bush et al. 2020), an increase in the frequency of water drinking (Wright et al. 2022), and range shifts for some species (Carvalho et al. 2021), to name a few.

The need for monitoring primate populations to have accurate estimates of changes over time seems obvious, but it is typically time-consuming, difficult, and expensive (Dobson et al. 2019; Kühl et al. 2008). Strier (2021) provides a valuable illustration of how anthropogenetic pressures are pushing primate populations to their limits of resilience as they face increasing threats such as disease, climate change, and habitat saturation. I have had the rare privilege of working with mountain gorillas, a subspecies that may be considered a conservation success story as the population size is estimated to have doubled over the past four decades (Robbins et al. 2011; Granjon et al. 2020; Hickey et al. 2019). Such a change was only possible through the efforts of many people and approaches, yet we cannot be complacent, given the multiple continued threats in a very limited habitat.

How can we move forward? Riley (2019) calls for an increase in interdisciplinary approaches to studying primate behavior as a means of better understanding primates living in human-influenced landscapes. Chapman et al. (2022) argue that the future of biodiversity, including primates, requires increased funding, increased research and education capacity in primate-range countries, as well as more studies that effectively influence policy decisions.

## Connections among people

Connections are invaluable for our social and professional lives. We all rely on students, colleagues, and staff for the successful running of research programs. Collaborative studies that enable cross-species comparisons within and between taxa are vital for understanding patterns of diversity in primate social behavior and life history patterns (e.g. Young and Robbins 2023; Kappeler et al. 2022; Colchero et al. 2021). Data collected at individual study sites for particular projects typically can be shared for comparative studies as well, such that the whole becomes greater than the sum of the parts.

**Fig. 2:**
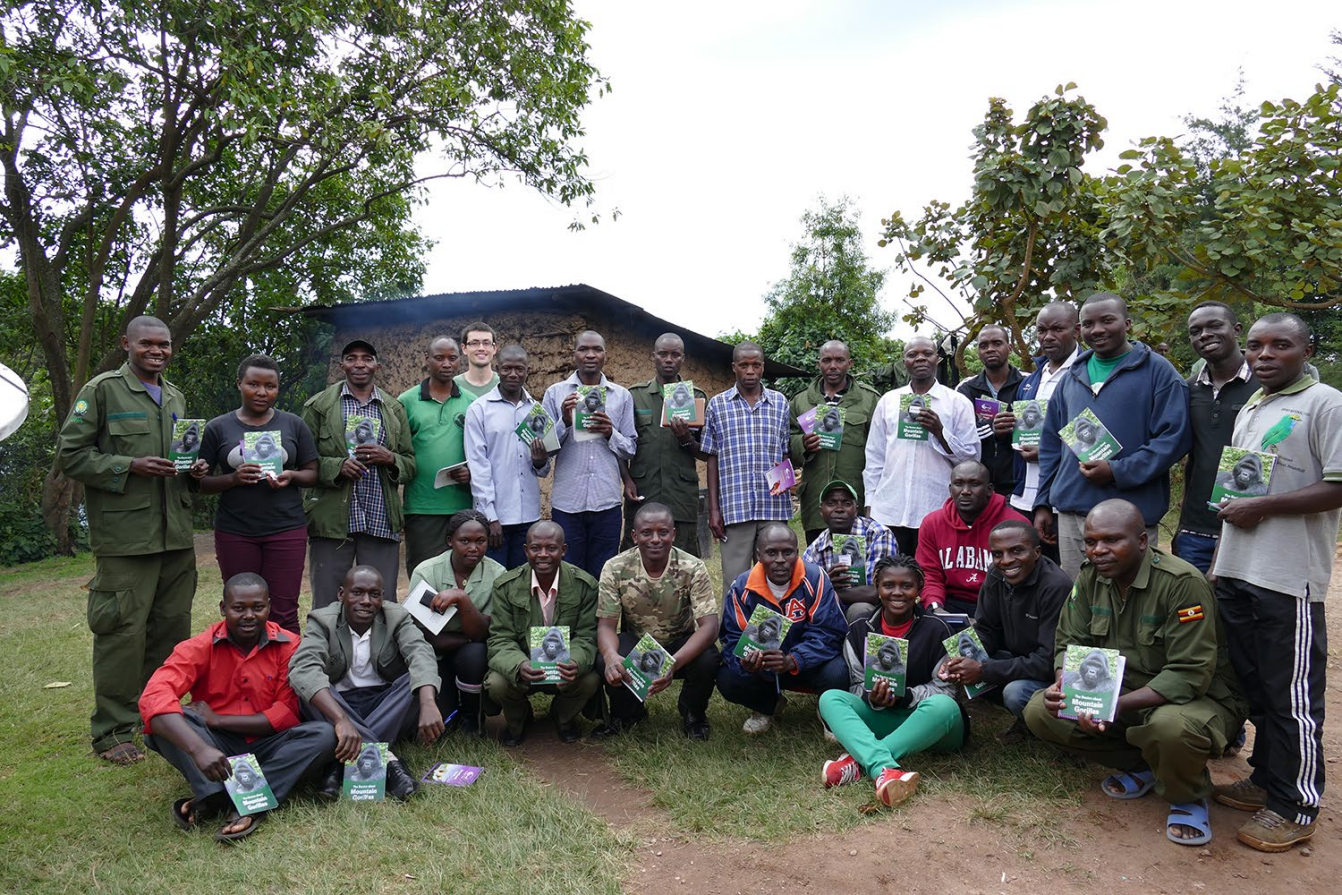
Group photo of a collaborative training between the Bwindi Gorilla Project and the Uganda Wildlife Authority

**Fig. 3:**
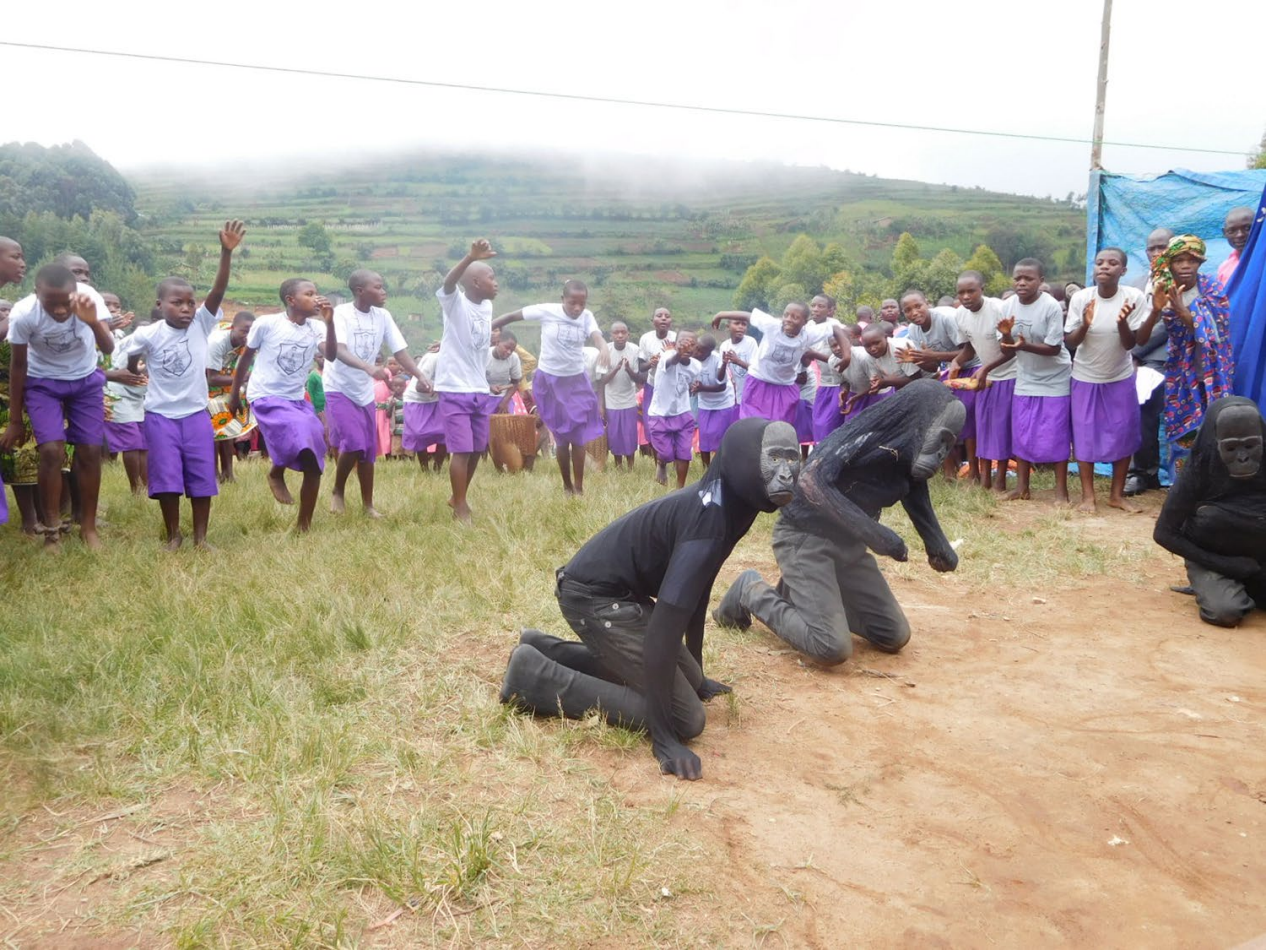
Primary school children enacting gorilla behavior during conservation education activities in Bwindi Impenetrable National Park, Uganda

The dissemination of scientific studies to a general audience is easier for primatologists than for many scientists due to how relatable primates and their social behavior are to the public. As discussed in the previous section, conservation is an increasingly vital component of most primate research projects. Collaborations among scientists, non-governmental organizations, government park services, and local communities can assist in scientific studies, training, information sharing, and community projects. My own research has involved working together with the Uganda Wildlife Authority to maintain a database of demographic information on all the habituated gorillas in Bwindi. This database has been used in scientific studies (e.g. Robbins et al. 2019), and, in turn, we assist in training the park staff about gorilla behavior and ecology through work in the forest and formal workshops (Fig. 2). Additionally, talking to local researchers, staff and community members about traditional and local views on primates and primate conservation provides valuable information (Riley 2019; Robbins 2021). Much work remains to be done in capacity building at all education levels (Chapman and Peres 2021; Chapman et al. 2022). Conservation education and community engagement are vital components of most primate field projects (Fig. 3), even if evidence of their impact on primate populations remains limited (Bettinger and Leighty 2021; Bettinger et al. 2021; Chapman and Peres 2021).

## An anecdote

I will end with an anecdote because there is often only room for them in editorials. I frequently reflect on two unrelated adult females, Binyindo and Siatu, who were in the main study group in Bwindi when I began the project in 1998. They both have been consistently high-ranking (Robbins 2008; Wright et al. 2014) but rarely affiliate with each other (only 30 observed grooming bouts between them in 25 years; nearly all of them from Siatu to Binyindo). Nonetheless, when the group fissioned in 2016, they remained in the same group. In 2017, they both transferred to a solitary male. Currently they co-reside with this silverback along with Binyindo’s daughter and grandson. Binyindo and Siatu have spent at least the past 25 years together, most of that time within 100 m of each other. That is some kind of social connection.

Binyindo and Siatu do not know that they serve as data points in scientific publications to further our understanding of primate social evolution, that they bring joy to thousands of tourists that visit them from all corners of the world, that they are endangered, or that people living in communities that neighbor their habitat face the challenges posed by poverty. However, from our perspective, they provide narratives that inspire us as scientists, conservationists, and citizens, on a local and international scale, to study and protect them, and other primate species as well, as we work to improve the livelihoods of their human neighbors.
